# The Relationship between Starting to Drink and Psychological Distress, Sleep Disturbance after the Great East Japan Earthquake and Nuclear Disaster: The Fukushima Health Management Survey

**DOI:** 10.3390/ijerph14101281

**Published:** 2017-10-24

**Authors:** Masatsugu Orui, Yuka Ueda, Yuriko Suzuki, Masaharu Maeda, Tetsuya Ohira, Hirooki Yabe, Seiji Yasumura

**Affiliations:** 1 Department of Public Health, Fukushima Medical University School of Medicine, Fukushima 960-1295, Japan; yrsuzuki@ncnp.go.jp (Y.S.); yasumura@fmu.ac.jp (S.Y.); 2 Department of Epidemiology, Fukushima Medical University School of Medicine, Fukushima 960-1295, Japan; yumu327@fmu.ac.jp (Y.U.); teoohira@fmu.ac.jp (T.O.); 3 Department of Adult Mental Health, National Institute of Mental Health, National Center of Neurology and Psychiatry, Tokyo 187-8553, Japan; 4 Department of Disaster Psychiatry, Fukushima Medical University School of Medicine, Fukushima 960-1295, Japan; masagen@fmu.ac.jp; 5 Radiation Medical Science Center for the Fukushima Health Management Survey, Fukushima Medical University School of Medicine, Fukushima 960-1295, Japan; hyabe@fmu.ac.jp; 6 Department of Neuropsychiatry, Fukushima Medical University School of Medicine, Fukushima 960-1295, Japan

**Keywords:** alcohol, evacuees, nuclear disaster, Great East Japan earthquake, mental health service

## Abstract

This longitudinal study aimed to investigate the prevalence of newly-started drinkers and their continuing drinking behaviors after the Great East Japan earthquake. Moreover, the relationships between newly-started drinking and psychological factor, disaster-related experience, and perceived radiation risk were examined. We used data from 37,687 pre-disaster non-drinkers who participated in the 2012 and 2013 surveys conducted in Fukushima. We defined newly-started drinkers as those who did not drink before the disaster but who began drinking after the disaster, based on information collected retrospectively. In 2012, 9.6% of non-drinkers began drinking, of which the prevalence of heavy drinkers was 18.4%. The prevalence of continued drinking among newly-started drinkers in 2013 was 53.8%. Logistic regression analyses revealed post-disaster newly-started drinking was significantly associated with being male, less than 65 years old, sleep dissatisfaction and psychological distress (Kessler 6 ≤ 13) when this model was adjusted for disaster-related experience and perceived radiation risk. Moreover, psychological distress and heavy drinking were significant risk factors for continued drinking among newly-started drinkers. Newly-started drinkers might use alcohol to cope with disaster-related stress. Thus, they may be targeted for disaster-related health services. Moreover, early intervention should encourage responsible drinking, since post-disaster heavy drinkers were likely to continue heavy drinking.

## 1. Introduction

The Great East Japan earthquake occurred on 11 March 2011, generating a massive tsunami and causing enormous damage to the Pacific Coast [1]. Later, a separate tsunami hit the Tokyo Electric Power Company’s Fukushima Daiichi Nuclear Power Plant and caused a radiation hazard in Fukushima Prefecture. This forced the long-term evacuation of residents from wide surrounding areas. The number of evacuees reached over 160,000 as of May 2012 [2]. This triple disaster—the earthquake, the tsunami, and the nuclear disaster—forced evacuees to face hardships, such as relocation to a non-hazardous area, separation of family members, loss of housing, as well as adjustment to new circumstances. These harsh experiences led to a very stressful situation for evacuees, consequently, could cause post-disaster mental distress, including post-traumatic stress disorder (PTSD), depression, and suicidality [3,4]. Studies have suggested that prompt mental health care is required when going through a catastrophe. For the Great East Japan earthquake, mental health care services are still being provided in the disaster-stricken areas [5,6].

Some disaster studies have reported that alcohol consumption increases following a disaster, due to psychological distress in affected individuals [7,8]. Furthermore, patients with post-traumatic stress disorder (PTSD) or alcohol use disorder often reported using alcohol to cope with post-traumatic stress [9,10,11,12,13,14,15]. Since the majority of evacuees in disaster-stricken areas are burdened with some stress related to disaster events, consequently, some evacuees may start drinking alcohol to cope with their stressful situation [16]. Therefore, we propose that early intervention or support is required for people who begin drinking alcohol in the context of disaster-related stress or difficulty in their life [17,18,19].

According to a report following the Hanshin-Awaji earthquake in Japan, many victims showed increased symptoms of stress, difficulties caused by anxiety and sleep disturbance, and depressive symptoms or hopelessness [20]. After the Great East Japan earthquake, evacuees have reported severe stress caused by socioeconomic issues in the long-term post-disaster phase, whereas significant effects on evacuees’ mental state from disaster-related experiences in the acute phase [21]. These findings suggest that the cause of evacuees’ stress may change with the passage of time. Therefore, because the evacuations are long-lasting, it is necessary to examine longitudinally how evacuees’ current physical/mental health status, and socioeconomic circumstances, is associated with newly-started drinking behavior.

In this study, we longitudinally investigated the prevalence of newly-started drinking and associated factors for two years after the Great East Japan earthquake. We also examined which factors are likely to pose a higher risk for continued drinking behavior among newly-started drinkers.

## 2. Materials and Methods

### 2.1. The Fukushima Health Management Survey

After the devastating triple disaster in Fukushima Prefecture, the annual Fukushima Health Management Survey began in 2011 in order to monitor evacuees’ health and lifestyle conditions. This survey was designed to determine whether the triple disaster affected the evacuees’ physical, psychological, and socioeconomic wellbeing [22]. We utilized data from the Mental Health and Lifestyle survey (conducted in 2012 and 2013), which is a part of the Fukushima Health Management Survey. This study was approved by the Committee for Ethics at Fukushima Medical University (No. 13020). The participants of the Mental Health and Lifestyle Survey were aged at least 15 years old and had lived in the evacuation zones specified by the national government: Tamura City, Minami-soma City, Kawamata Town, Hirono Town, Naraha Town, Tomioka Town, Kawauchi Village, Okuma Town, Futaba Town, Namie Town, Katsurao Village, and Iitate Village. The size of the original target cohort was 180,604 as of 31 March 2011. Residents who were living in an evacuation area on 11 March 2011 and had experienced evacuation to the non-hazardous area, received the survey questionnaire. In order to address non-respondent bias, and to raise response rate, sending a reminder or performing public relation activities were implemented. The number of respondents was 70,193 (Rate: 38.9%) in the first survey conducted in 2012, and 54,890 (Rate: 30.4%) in the second survey conducted in 2013.

### 2.2. Participants

**Figure 1:**
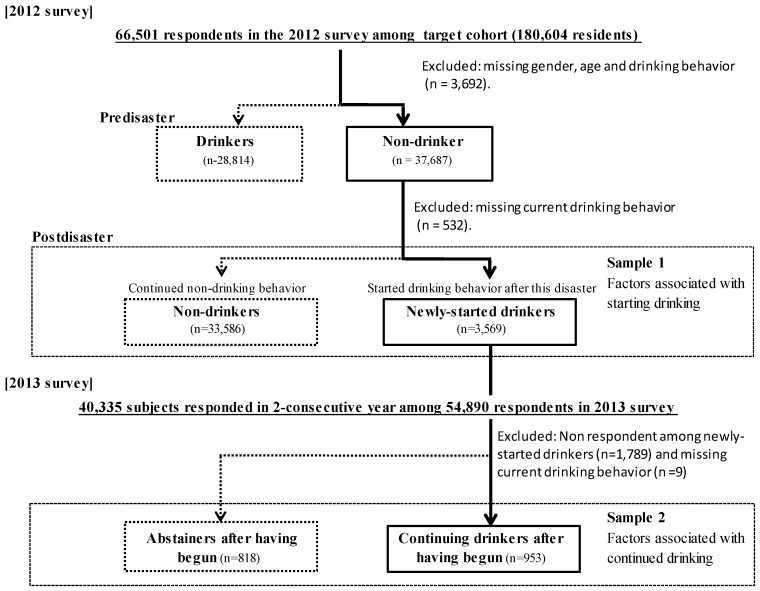
Study sample.

For the current study, we used data from respondents in the 2012 and 2013 surveys who were at least 20 years old on 11 March 2011, and who reported in the 2012 survey that they were a non-drinker before the disaster. Among the 66,501 respondents in the 2012 survey, 37,687 of pre-disaster non-drinkers were analyzed in order to evaluate factors associated with post-disaster newly-started drinking (Figure 1).

Among 180,604 residents in the target cohort, 66,501 responded to 2012 survey. After excluding the subjects missing gender, age and drinking behavior, 37,687 respondents of non-drinkers pre-disaster was analyzed. Of this group, 3569 evacuees began drinking post-disaster, including 2913 light drinkers and 656 heavy drinkers. Among these newly-started drinkers, 953 evacuees continued drinking, including 227 heavy drinkers, in the 2013 survey.

### 2.3. Classification of Drinking Behavioral Pattern

First of all, we grasped non-drinkers in pre-disaster by self-reporting in closed questionnaire. Respondents in the 2012 survey self-reported their drinking habit in pre-disaster as “don’t drink or drinking only rarely (less than once per month),” and “current drinker (at least once per month).” Moreover, they answered their current alcohol consumption as “don’t drink or drinking only rarely (less than once per month),” “quit (history of drinking but not a current drinker),” and “current drinker (at least once per month) in the same survey conducted in 2012. Furthermore, we investigated the same questionnaire in the 2013 survey and grasped the respondents’ current drinking behavior (Don’t drink or drinking only rarely, quit and current drinkers).

By these procedures, we defined newly-started drinkers and continuing drinkers after having begun. “Newly-started drinkers (having drinking behavior at least once per month) after this disaster” were as those who reported that they did not drink or drinking only rarely before the disaster and were a current drinker in the 2012 survey. Newly-started drinkers who reported being a current drinker in both the 2012 and 2013 surveys were labelled as “continuing drinkers (having drinking behavior at least once per month)”.

Moreover, drinkers were further categorized into heavy drinking and light drinking groups, based on the amount of alcohol consumed on a typical drinking day. We defined heavy drinkers as those who consumed four drinks per day and more, and light drinkers as those who consumed four or less drinks per day. The reason was that the National Health Promotion of Japanese Ministry of Health, Labor and Welfare defined the heavy drinking as 6 Drinks per day in only on the days of drinking among both genders. However, this definition of heavy drinking might be a large amount for newly-started drinkers. Therefore, we utilized the cutoff point as “4 drinks per day” in only on the days of drinking, which is based on recommended limits of drinking in male (4 Drinks per day) in the National Health Promotion. By the way, 4-Drrink was defined as 120 mL of spirits (e.g., whiskey or brandy), 480 mL of wine, 1000 mL of beer, or 360 mL of Japanese sake.

### 2.4. Measurements

The Mental Health and Lifestyle Survey mainly assesses lifestyle factors (diet, exercise, sleep, and smoking or drinking behaviors), disaster-related experiences, socioeconomic circumstances, general subjective health status, current mental health status, and perceived risks from radiation exposure [22]. In order to evaluate the risk factors associated with newly-started drinking, we categorized the items in the Mental Health and Lifestyle Survey as follows: (1) current physical/mental health status (general subjective health status, sleep disturbance, and Kessler 6-item scale (K6)); (2) disaster-related experience (experience of tsunami or nuclear power plant accident); (3) effects of the disaster on socioeconomic status (loss of employment due to disaster); and (4) perceived risk of radiation exposure. Kessler 6-item scale (K6) and perceived risk of radiation exposure are validated measurements and the others are investigator-designed queries. Additionally, to assess factors associated with continued drinking, we utilized drinking behavior in the 2012 survey (heavy or light drinkers) as an adjustment variable.

#### 2.4.1. Current Physical/Mental Health Status

To evaluate the relationship between health status and drinking behavior, general subjective health status and dissatisfaction with sleep were measured on a Likert scale (general subjective health status: 1 = ‘very well’ to 5 = ‘very poor’; satisfaction with sleep: 1 = ‘satisfied’ to 4 = ‘strongly dissatisfied’). We defined those reporting ‘poor’ and ‘very poor’ general subjective health status as the ‘poor health’ group. Those who responded that their level of satisfaction with their sleep was ‘slightly dissatisfied’, ‘dissatisfied’, or ‘strongly dissatisfied’ were assigned to the ‘dissatisfied with sleep’ group.

To assess mental health status among residents, we utilized the K6 scale to screen for non-specific psychological distress [23]. Those scoring 0–12 points were classified as having probable mild–moderate/probable no psychological distress and those scoring 13–24 points were classified as having probable severe psychological distress [23]. This study used the Japanese version of the K6, which has been empirically validated as an independent means of screening for psychological distress among evacuees [24].

#### 2.4.2. Disaster-Related Experience

Disaster-related experiences, including experience of the tsunami and nuclear power plant accident, were utilized as adjustment factors. In this survey, the experience of nuclear power plant accident was categorized according to whether respondents heard an explosion or not. It was only natural that the experience of the earthquake would be an important disaster-related experience in this triple disaster; however, the vast majority of respondents to the Fukushima Health Management Survey have shared this experienced. For this reason, we excluded experience of the earthquake from our analyses.

#### 2.4.3. Effects of the Disaster on Socioeconomic Status

It has been reported that socioeconomic circumstances may affect evacuees’ psychological status [25]. Thus, it was essential to assess the association between evacuees’ socioeconomic status and alcohol consumption. In this study, we utilized the variable ‘loss of employment due to disaster’ as indicating socioeconomic status.

#### 2.4.4. Perceived Risk of Radiation Exposure

Radiation exposure in the triple disaster was an unprecedented experience among the evacuees, and their perceived radiation exposure risk may have affected their disaster-related stress or psychological distress. We measured participants’ perceived risk of radiation exposure with the following question: “What do you think is the likelihood of damage to your health (e.g., cancer onset) in later life as a result of your current level of radiation exposure?” [26]. This question was answered on a four-point Likert scale: 1 = ‘very unlikely’ to 4 = ‘very likely’. In this study, we combined ‘very unlikely’ and ‘unlikely’ into the ‘low-perceived risk’ group. Likewise, ‘likely’ and ‘very likely’ were combined into the ‘high-perceived risk’ group.

### 2.5. Statistical Analysis

Firstly, we excluded subjects who were missing age, gender, drinking behavior in pre-disaster and current before analyses (Figure 1). To assess factors associated with newly-started drinking in the 2012 survey, and with continued or discontinued drinking among newly-started drinkers in the 2013 survey, we performed chi-square tests and multivariate logistic analyses with psychological, socioeconomic, disaster-related experience, and perceived radiation risk as independent variables. Besides, we also performed a multivariate logistic regression analysis on continued drinking in the 2013 survey, adjusted by heavy/non-heavy drinking behavior in 2012 survey (previous heavy drinking behavior was assumed to be an important risk factor for continued drinking).

Statistical significance was evaluated using two-sided, design-based tests with a *p* < 0.05 level of significance. All statistical analyses were performed using IBM SPSS Statistics version 23.0 (IBM Corp., Armonk, NY, USA).

## 3. Results

### 3.1. The Prevalence of Newly-Started Drinking Behavior and Continued Drinking after the Disaster

The characteristics of respondents in the 2012 survey (including pre-disaster drinkers and non-drinkers) are shown in Table 1. There were a higher proportion of females and those aged 65 years and older in the non-drinking group than the drinking group.

**Figure 2:**
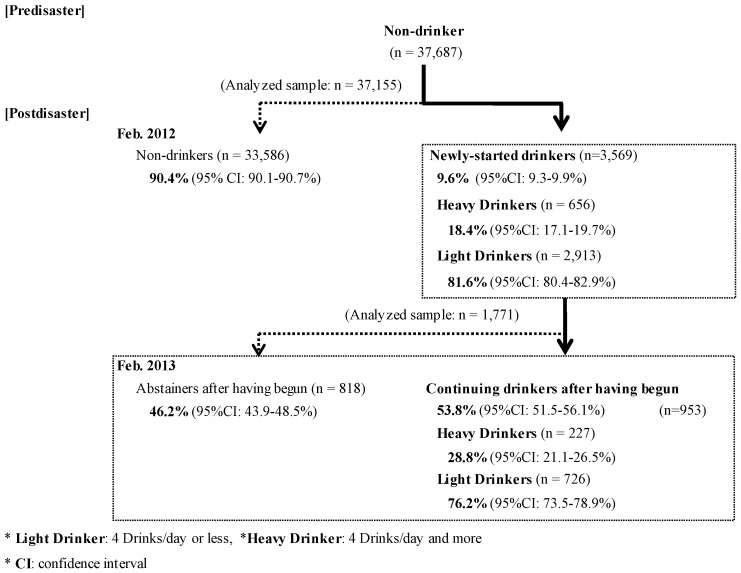
Prevalence of newly-started drinkers and continuing drinkers after having begun.

Among non-drinkers before the disaster, 3569 evacuees (9.6%, 95% CI (confidence interval): 9.3–9.9%) reported that were current drinkers in the 2012 survey, of which 656 (18.4%, 95% CI: 17.2–19.7%) were heavy drinkers. Among these newly-started drinkers, 953 respondents (53.8%, 95% CI: 51.5–56.1%) continued drinking at the time of the 2013 survey. The number of heavy drinkers was 227 (23.8%, 95% CI: 21.1–26.5%) in 2013 (Figure 2).

Among the 2012 survey respondents, the prevalence of non-drinkers pre-disaster was 9.6% (95% CI: 9.3–9.9%). Of this group, heavy drinkers who have begun drinking post-disaster was 18.4% (95% CI: 17.1–19.7%), and light drinkers was 81.6% (95% CI: 80.4–82.9%). Among these newly-started drinkers, 53.8% (95% CI: 51.5–56.1%) of them continued drinking. Moreover, the prevalence of heavy and light drinkers in this group were 28.8% (95% CI: 21.1–26.5%), 72.2% (95% CI: 73.5–78.9%), respectively, in the 2013 survey.

### 3.2. Factors Related to Newly-Started Drinking Behavior

Table 2 shows the distribution of 2012 newly-started drinkers’ and non-drinkers’ characteristics, disaster-related experiences, economic factors, general subjective health status, dissatisfaction with sleep condition, psychological distress (K6), and radiation risk perception. In a chi-square test, newly-started drinking behavior was greater among males, younger generations (the ages of 20–39 and 40–64 years old), and those with higher education (Vocational college, University, Graduated school). The proportion of newly-started drinkers with disaster-related experience, unemployment due to disaster, psychological distress, and perception of radiation risks was much higher than the reference population. Among newly-started drinkers, heavy drinking was associated with being male, younger (the ages of 20–39 and 40–64 years old), higher education, dissatisfaction with sleep, and psychological distress.

We performed a multivariate logistic regression to examine factors related to post-disaster newly-started drinking in the 2012 survey (Table 3). In Model 1, we analyzed the association between current physical/mental health status and newly-started drinking. Dissatisfaction with sleep (Odds ratio [OR]: 1.08, 95% CI: 1.05–1.11, *p* < 0.01) and psychological distress (OR: 1.12, 95% CI: 1.09–1.15, *p* < 0.01) were significantly associated with newly-started drinking. General subjective health status was not a significant risk factor (OR: 0.96. 95% CI: 0.94–0.98, *p* < 0.01). Model 2 was adjusted by disaster-related experience, the effect of the disaster on socioeconomic status, and perception of radiation risks. Newly-stassrted drinking in 2012 was significantly associated with being male (OR: 1.95, 95% CI: 1.78–2.13, *p* < 0.01), aged under 40 years old (OR: 2.30, 95% CI: 2.03–2.60, *p* < 0.01), dissatisfaction with sleep (OR: 1.07, 95% CI: 1.05–1.10, *p* < 0.01), psychological distress (OR: 1.11, 95% CI: 1.08–1.14, *p* < 0.01), whose findings were not significantly different with the Model 1 results. General subjective “poor health” status was not a risk factor for newly-started drinking (OR: 0.96, 95% CI: 0.93–0.98, *p* < 0.01). In a comparison between heavy and light drinkers in Model 3, those who had been suffered severe psychological distress were more likely to drink heavily (OR: 1.14, 95% CI: 1.07–1.22, *p* < 0.01).

### 3.3. Factors Related to Continued Drinking among Newly-Started Drinkers

Table 4 provides the distribution of the characteristics of those who reported newly-started drinking in 2012 and reported either continued or discontinued drinking in 2013. Also, the distribution of heavy and light drinkers is shown. Being male, younger, and a heavy drinker in 2012 were all significantly associated with continued drinking in the 2013 survey.

We performed a multivariate logistic regression to examine factors associated with continued drinking in 2013. In Model 2 (adjusted for disaster-related experience, effects of disaster on socioeconomic status, perception of radiation risks, and previous drinking behavior), psychological distress (OR: 1.10, 95% CI: 1.00–1.20, *p* = 0.04) and heavy drinking behavior in 2012 (OR: 1.21, 95% CI: 1.12–1.31, *p* < 0.01) were significantly associated with continued drinking in 2013, as with results in Model 1 (Table 5).

## 4. Discussion

### 4.1. Factors Related to Newly-Started Drinking Behavior and Continued Drinking among Newly-Started Drinkers

Our findings from the 2012 Fukushima Health Management Survey showed that newly-started drinking was significantly associated with sleep dissatisfaction and psychological distress. Moreover, psychological distress and heavy drinking were significant risk factors for continued drinking among newly-started drinkers. It may indicate that some evacuees used alcohol to self-medicate their sleep problems and psychological distress.

These findings are similar to previous reports. In the 2004 Southeast Asia tsunami disaster, severe exposure to the tsunami was associated with self-reported increased alcohol consumption [8]. In a previous study, patients with post-traumatic stress disorder (PTSD) or alcohol use disorder often reported using alcohol to cope with post-traumatic stress [9,10,11,12,13,14,15]. Thus, newly-started drinkers who use alcohol to cope with stressful situations may constitute an appropriate group to target for disaster-related mental health services [27].

We proposed that the factors associated with beginning to drink alcohol in the post-disaster period could be different from those associated with continued drinking among newly-started drinkers. This is because the evacuees’ subjective health status, and their socioeconomic condition, may change over time due to the long period of evacuation. Our results showed that psychological distress (K6 score ≥13 points) was significantly associated with continued drinking, whereas sleep dissatisfaction and disaster-related experiences were not significantly associated. Extended observation, therefore, could give different findings; those who have continued to drink while experiencing psychological distress might be more affected by the long-term changes in socioeconomic status or stressful lifestyle under evacuation [20,21].

However, there may be different factors associated with newly-started drinking among young people (who are permitted to drink alcohol at age 20, according to Japanese laws) compared to the middle-aged and elderly people who were affected by this accident. Therefore, we analyzed a subset of respondents who were over 40 years old. However, this did not show a significant effect in the 2012 and 2013 surveys (shown in Supplementary Materials).

In our findings, heavy drinking behavior (four drinks per day and more) among newly-started drinkers in 2012 was a significant risk factor for continued drinking behavior in 2013. Therefore, those who begin drinking post-disaster, especially those who drink heavily, should be high-priority subjects for disaster-related mental health services. By following up on their health condition and, if necessary, providing early intervention to encourage responsible drinking, it may be possible to prevent heavy drinking and continued drinking behavior [17,18,19].

Otherwise, disaster-related experience, disaster effects on economic status (loss of employment due to disaster) and perception of radiation risks did not have a stronger association with starting to drink behavior or continuing drinking behavior, compared with psychological distress. This was because these odds ratios of these variables were relatively small. The effects of the disaster on socioeconomic statuses, such as loss of employment, was hypothesized to be a risk factor for post-disaster newly-started drinking and continued drinking [28]. It is thought that these factors could cause deterioration in economic status or working conditions, which affects mental health [26,29]. According to our analysis, however, loss of employment was unlikely to be a risk factor for continued drinking in the 2013 survey, whereas it was likely a risk factor for newly-started drinking in the 2012 survey. The reason for this difference is unclear. However, those who lived in the evacuation areas were given compensation for mental damage, loss of income, damage to the agriculture, forestry, and fishery industries, and damage to the food industry caused by harmful rumors after the nuclear disaster [30]. Therefore, financial aid given to evacuees might have mitigated the effect of loss of employment on continued drinking.

Due to the accident at the nuclear power plant, we considered that perception of radiation risk would have an impact on evacuees’ drinking behavior. According to a previous report regarding radiation risk perception among evacuees in Fukushima Prefecture, those who perceived a very high risk from radiation exposure tended to have psychological distress [31]. Our findings showed that the perception of health risks from radiation exposure had a significant association with newly-started drinking in the 2012 survey, whereas there was no significant association with continued drinking in the 2013 survey. Our finding could be explained by the weakening of perceived risks over time (between the 2012 to 2013 surveys, the proportion of those who perceived a high risk of radiation decreased from 47.7% to 39.4% among all respondents; data not shown).

### 4.2. Limitations and Strengths

The present study has several limitations. First, the response rate was less than 40% in the 2012 and 2013 survey. Therefore, the results of this study may not generalize to all evacuees within the areas specified by the government. Moreover, previous studies showed that the mental health status might have effects on response rate to survey, suggesting that non-response was associated with bad mental health status [32,33]. There might be many evacuees who were in bad condition and could not answer the survey, which might be underestimated in our findings.

Second, there was inadequate information on physical health status in the present study, such as the results of health checks. Although a history of the physical disease may affect drinking behaviors, the information on physical disease history could include some information bias because of self-reporting. Therefore, general subjective health status was utilized as a variable of physical health status even though it may be an insufficient variable. Finally, since there is a lack of information regarding the amount of alcohol consumed before the disaster, we could not examine change in the amount of alcohol consumed in the pre- and post-disaster periods. Furthermore, recall bias should be considered because we conducted these survey after this disaster by self-reporting, and collected pre-disaster drinking status retrospectively without validated measures such as AUDIT (the Alcohol Use Disorders Identification Test). This is an important limitation of this study.

Despite these limitations, this study has multiple strengths. Studies regarding the association between alcohol consumption and disasters have been conducted using longitudinal analysis which determined the association between drinking behaviors and related factors. This was a large-scale survey of the evacuees who were residing within the designated evacuation areas in Fukushima; it can be utilized to assist evacuees in recovering from mental health damage. It is anticipated that psychological and socioeconomic issues related to newly-started drinking among evacuees will be taken into account in ongoing mental health service activities.

## 5. Conclusions

Our findings showed that newly-started drinking was associated with being male, younger, dissatisfaction with sleep, and psychological distress following the disaster. Heavy drinking and psychological distress were risk factors for continued drinking among newly-started drinkers. It should be noted that newly-started drinkers who use alcohol to cope with disaster-related stress may be a suitable target group for disaster-related mental health services. Furthermore, it is essential to encourage responsible drinking and promote early intervention for heavy drinkers, since our findings show that newly-started drinkers who drink heavily are more likely than light drinkers to continue drinking. We hope our work will have implications for disaster-related mental health service providers.

## Figures and Tables

**Table 1:** Demographic characteristics of respondents.

	Pre-Disaster				*p* Value (χ^2^)
	Drinkers		Non-Drinkers		
	(n = 28,814)		(n = 37,687)		
	n	(%)	n	(%)	
**Gender**					
Male	19,063	(66.2)	10,567	(28.0)	<0.01 (χ^2^ = 9605.3)
Female	9751	(33.8)	27,120	(72.0)	
**Age (as of 11 March 2011)**					
20–39 years	6755	(23.5)	7766	(20.8)	
40–64 years	14,342	(49.9)	14,642	(39.2)	<0.01 (χ^2^ = 1329.9)
65 years and older	7628	(26.6)	14,907	(39.9)	
**Education**					
Junior/Senior high school	20,098	(71.9)	27,645	(76.7)	<0.01 (χ^2^ = 190.6)
Vocational college, University, Graduated school	7865	(28.1)	8415	(23.3)	

**Table 2:** Characteristics of newly-started drinkers and continuing non-drinkers in the 2012 survey.

		Total				*p* Value (χ^2^)	Newly-Started Drinkers				*p* Value (χ^2^)
		Newly-Started Drinkers		Continuing Non-Drinkers			Heavy Drinkers		Light Drinkers		
		(n = 3569)		(n = 33,593)			(n = 656)		(n = 2913)		
		n	(%)	n	(%)		n	(%)	n	(%)	
**Gender**											
Male		1506	(42.2)	8978	(26.7)	<0.01 (χ^2^ = 381.0)	375	(57.2)	1131	(38.8)	<0.01 (χ^2^ = 73.8)
Female		2063	(57.8)	24,608	(73.3)		281	(42.8)	1782	(61.2)	
**Age (as of 11 March 2011)**											
20–39 years old		1092	(31.7)	6667	(20.0)		304	(27.8)	788	(27.9)	
40–64 years old		1397	(40.5)	13,116	(39.3)	<0.01 (χ^2^ = 332.7)	251	(23.0)	1146	(40.6)	<0.01 (χ^2^ = 145.4)
65 years old and older		960	(27.8)	13,553	(40.7)		70	(6.4)	890	(31.5)	
**Education**											
Junior/Senior high school		2433	(70.5)	24,819	(77.2)	<0.01 (χ^2^ = 78.3)	410	(64.5)	2023	(71.8)	<0.01 (χ^2^ = 13.5)
Vocational college, University, Graduated school		1020	(29.5)	7339	(22.8)		226	(35.5)	794	(28.2)	
**General subjective health status**											
Poor		691	(19.8)	7138	(21.8)	0.01 (χ^2^ = 7.42)	450	(78.5)	1650	(71.2)	<0.01 (χ^2^ = 12.4)
Good/Unremarkable		2803	(80.2)	25,650	(78.2)		123	(21.5)	667	(28.8)	
**Sleep disturbance**											
Unsatisfied with sleep condition	Yes	2100	(72.7)	17,965	(66.5)	<0.01 (χ^2^ = 45.0)	450	(78.5)	1650	(71.2)	<0.01 (χ^2^ = 12.4)
	No	790	(27.3)	9051	(33.5)		123	(21.5)	667	(28.8)	
**Psychological distress**											
K6 (13 points and more)	13≤	670	(20.3)	4942	(15.8)	<0.01 (χ^2^ = 43.4)	178	(28.2)	492	(18.4)	<0.01 (χ^2^ = 30.1)
	≤12	2634	(79.7)	26,273	(84.2)		454	(71.8)	2180	(81.6)	
**Disaster-related experience**											
Experience of tsunami	Yes	771	(21.6)	6179	(18.4)	<0.01 (χ^2^ = 21.8)	149	(22.7)	622	(21.4)	0.44 (χ^2^ = 0.59)
	No	2798	(78.4)	27,407	(81.6)		507	(77.3)	2291	(78.6)	
Experience of nuclear power plant accident	Yes	1969	(55.2)	17,267	(51.4)	<0.01 (χ^2^ = 18.3)	394	(60.1)	1575	(54.1)	0.01 (χ^2^ = 7.78)
	No	1600	(44.8)	16,319	(48.6)		262	(39.9)	1338	(45.9)	
**Disaster effects on economic status**											
Loss of employment due to disaster	Yes	823	(23.1)	6420	(19.1)	<0.01 (χ^2^ = 32.0)	158	(24.1)	665	(22.8)	0.49 (χ^2^ = 0.48)
	No	2746	(76.9)	27,166	(80.9)		498	(75.9)	2248	(77.2)	
	No	11,018	(91.6)	1012	(8.4)		833	(82.3)	179	(17.7)	
**Perception of radiation risks**											
Delayed effects	Low	2781	(82.6)	26,498	(85.1)		512	(80.1)	2269	(83.2)	
	High	1811	(53.7)	14,925	(47.8)	<0.01 (χ^2^ = 43.5)	376	(58.7)	1435	(52.6)	0.01 (χ^2^ = 6.53)

Notes: Light Drinker: 4 Drinks/day or less; Heavy Drinker: 4 Drinks/day and more; K6: Kessler 6-item scale.

**Table 3:** Multivariate logistic regression analysis of newly-started drinkers (2012).

		Model 1			Model 2			Model 3		
		Newly-Started Drinkers vs. Continued Non-Drinking in 2012 Survey						Newly-Started Drinkers		
		Adjusted by Current Physical/Mental Health Status			Adjusted by Disaster-Related Experience, Disaster Effects on Economic Status and Perception of Radiation Risk			Heavy vs. Light Drinkers		
		Odds Ratio (95% CI)		*p* Value	Odds Ratio (95% CI)		*p* Value	Odds Ratio (95% CI)		*p* Value
**Gender**										
Male		1.99	(1.83–2.17)	<0.01	1.95	(1.78–2.13)	<0.01	2.56	(2.07–3.16)	<0.01
Female (Ref.)		1.00			1.00			1.00		
**Age (as of 11 March 2011)**										
20–39 years		2.26	(2.01–2.55)	<0.01	2.30	(2.03–2.60)	<0.01	5.24	(3.62–7.59)	<0.01
40–64 years		1.46	(1.33–1.61)	<0.01	1.46	(1.32–1.61)	<0.01	1.72	(1.38–2.15)	<0.01
65 years and older (Ref.)		1.00			1.00			1.00		
**Education**										
Junior/Senior high school		0.96	(0.94–0.98)	<0.01	0.96	(0.94–0.99)	<0.01	0.97	(0.92–1.03)	0.35
Vocational college, University, Graduated school (Ref.)		1.00			1.00			1.00		
**General subjective health status**										
Poor		0.96	(0.93–0.98)	0.01	0.96	(0.93–0.98)	<0.01	1.02	(0.95–1.10)	0.51
Good/Unremarkable (Ref.)		1.00			1.00			1.00		
**Sleep disturbance**										
Dissatisfied with sleep condition	Yes	1.08	(1.05–1.11)	<0.01	1.07	(1.05–1.10)	<0.01	1.07	(1.00–1.15)	0.04
	No	1.00			1.00			1.00		
**Psychological distress**										
K6 (13 points and more)	13≤	1.12	(1.09–1.15)	<0.01	1.11	(1.08–1.14)	<0.01	1.14	(1.07–1.22)	<0.01
	≤12	1.00			1.00			1.00		
**Disaster–related experience**										
Experience of tsunami	Yes	–	–	–	1.04	(1.01–1.07)	0.01	1.00	(0.94–1.07)	0.99
	No	–			1.00			1.00		
Experience of nuclear power plant accident	Yes	–	–	–	1.03	(1.00–1.05)	0.03	1.07	(1.02–1.13)	0.01
	No	–			1.00			1.00		
**Disaster effects on economic status**										
Loss of employment due to disaster	Yes	–	–	–	1.02	(0.99–1.05)	0.12	0.97	(0.91–1.03)	0.27
	No	–			1.00			1.00		
**Perception of radiation risks**										
Delayed effects	High				1.04	(1.02–1.06)	<0.01	1.00	(0.95–1.05)	0.97
	Low				1.00			1.00		

Notes: Light Drinker: 4 Drinks/day or less; Heavy Drinker: 4 Drinks/day and more; K6: Kessler 6-item scale.

**Table 4:** Characteristics of newly-started drinkers who continued drinking in the 2013 survey.

		2013 Drinking Status among Newly-Started Drinkers				*p* Value (χ^2^)	Continued Drinking				*p* Value (χ^2^)
		Continued Drinking		Discontinued Drinking			Heavy Drinkers		Light Drinkers		
		(n = 953)		(n = 818)			(n = 227)		(n = 726)		
		n	(%)	n	(%)		n	(%)	n	(%)	
**Gender**											
Male		398	(41.8)	286	(35.0)	<0.01 (χ^2^ = 8.56)	124	(54.6)	274	(37.7)	<0.01 (χ^2^ = 20.3)
Female		555	(58.2)	532	(65.0)		103	(45.4)	452	(62.3)	
**Age (as of 11 March 2011)**											
20-39 years		267	(28.6)	229	(28.5)		83	(37.9)	184	(25.8)	
40-64 years		434	(46.5)	313	(39.0)	<0.01 (χ^2^ = 14.6)	103	(47.0)	331	(46.4)	<0.01 (χ^2^ = 19.7)
65 years and older		232	(24.9)	261	(32.5)		33	(15.1)	199	(27.9)	
**Education**											
Junior/Senior high school		610	(66.6)	548	(69.9)	0.15 (χ^2^ = 2.12)	129	(60.3)	481	(68.5)	0.03 (χ^2^ = 5.00)
Vocational college, University, Graduated school		306	(33.4)	236	(30.1)		85	(39.7)	221	(31.5)	
**General subjective health status**											
Poor		175	(19.1)	155	(19.5)	0.82 (χ^2^ = 0.05)	53	(23.9)	122	(17.5)	0.04 (χ^2^ = 4.39)
Good/ Unremarkable		743	(80.9)	640	(80.5)		169	(76.1)	574	(82.5)	
**Sleep disturbance**											
Dissatisfied with sleep condition	Yes	644	(69.3)	525	(66.5)	0.22 (χ^2^ = 1.52)	168	(75.7)	476	(67.3)	0.02 (χ^2^ = 5.54)
	No	285	(30.7)	264	(33.5)		54	(24.3)	231	(32.7)	
**Psychological distress**											
K6 (13 points and more)	13≤	141	(15.6)	90	(11.7)	0.02 (χ^2^ = 5.35)	43	(19.6)	98	(14.3)	0.06 (χ^2^ = 3.64)
	≤12	765	(84.4)	682	(88.3)		176	(80.4)	589	(85.7)	
**Disaster-related experience**											
Experience of tsunami	Yes	205	(21.5)	166	(20.3)	0.53 (χ^2^ = 0.39)	58	(25.6)	147	(20.2)	0.09 (χ^2^ = 2.88)
	No	748	(78.5)	652	(79.7)		169	(74.4)	579	(79.8)	
Experience of nuclear power plant accident	Yes	863	(90.6)	725	(88.6)	0.18 (χ^2^ = 1.76)	210	(92.5)	653	(89.9)	0.25 (χ^2^ = 1.33)
	No	90	(9.4)	93	(11.4)		17	(7.5)	73	(10.1)	
**Effect of disaster on economic status**											
Loss of employment due to disaster	Yes	241	(25.3)	216	(26.4)	0.59 (χ^2^ = 0.29)	64	(28.2)	177	(24.4)	0.25 (χ^2^ = 1.33)
	No	712	(74.7)	602	(73.6)		163	(71.8)	549	(75.6)	
**Perception of radiation risks**											
Delayed effects	Low	486	(54.3)	451	(58.4)	0.09 (χ^2^ = 2.86)	107	(48.9)	379	(56.1)	0.06 (χ^2^ = 3.46)
	Low	416	(46.7)	362	(47.2)		90	(41.1)	326	(148.9)	
**Drinking behavior in 2012**											
Heavy drinkers (4 drinks/day and more)		216	(22.7)	90	(11.0)	<0.01 (χ^2^ = 41.9)	125	(55.1)	91	(12.5)	<0.01 (χ^2^ = 178.5)
Light drinkers (less than 4 drinks/day)		737	(77.3)	728	(89.0)		102	(44.9)	635	(87.5)	

Notes: Light Drinker: 4 Drinks/day or less; Heavy Drinker: 4 Drinks/day and more; K6: Kessler 6-item scale.

**Table 5:** Multivariate logistic regression analysis of continued drinking (2013).

		Model 1			Model 2			Model 3		
		Continued vs. Discontinued Drinking						Continued Drinking		
		Adjusted by Current Physical/Mental Health Status			Adjusted by Disaster–Related Experience, Disaster Effects on Economic Status, Perception of Radiation Risk and Heavy Drinking			Heavy vs. Light Drinkers		
		Odds Ratio (95% CI)		*p* Value	Odds Ratio (95% CI)		*p* Value	Odds Ratio (95% CI)		*p* Value
**Gender**										
Male		1.38	(1.12–1.71)	<0.01	1.22	(0.97–1.53)	0.08	1.84	(1.25–2.72)	<0.01
Female (Ref.)		1.00			1.00			1.00		
**Age (as of 11 March 2011)**										
20–39 years		1.20	(0.90–1.61)	0.22	1.01	(0.74–1.37)	0.97	1.74	(0.96–3.15)	0.07
40–64 years		0.78	(0.61–0.99)	0.05	0.69	(0.53–0.89)	0.01	1.18	(0.76–1.81)	0.46
65 years and older (Ref.)		1.00			1.00			1.00		
**Education**										
Junior/Senior high school		0.97	(0.92–1.03)	0.29	0.96	(0.91–1.02)	0.20	0.93	(0.84–1.03)	0.15
Vocational college, University, Graduated school (Ref.)		1.00			1.00			1.00		
**General subjective health status**										
Poor		0.96	(0.90–1.04)	0.31	0.96	(0.89–1.04)	0.31	1.04	(0.91–1.19)	0.54
Good/Unremarkable (Ref.)		1.00			1.00			1.00		
**Sleep disturbance**										
Dissatisfied with sleep condition	Yes	1.02	(0.96–1.08)	0.57	1.00	(0.95–1.07)	0.92	1.02	(0.92–1.14)	0.69
	No	1.00			1.00			1.00		
**Psychological distress**										
K6 (13 points and more)	13≤	1.09	(1.00–1.19)	0.04	1.10	(1.00–1.20)	0.04	1.06	(0.92–1.22)	0.39
	≤12	1.00			1.00			1.00		
**Disaster–related experience**										
Experience of tsunami	Yes	–	–	–	1.02	(0.95–1.09)	0.56	1.10	(0.98–1.23)	0.10
	No	–			1.00			1.00		
Experience of nuclear power plant accident	Yes	–	–	–	1.06	(0.97–1.16)	0.22	1.00	(0.83–1.20)	0.97
	No	–			1.00			1.00		
**Effect of disaster on economic status**										
Loss of employment due to disaster	Yes	–	–	–	0.95	(0.89–1.01)	0.07	1.07	(0.96–1.19)	0.23
	No	–			1.00			1.00		
**Perception of radiation risks**										
Delayed effects	High	–	–	–	1.03	(0.97–1.09)	0.34	1.07	(0.97–1.18)	0.19
	Low	–			1.00			1.00		
**Drinking behavior in 2012**										
Heavy drinkers (4 drinks/day and more)		–	–	–	1.21	(1.12–1.30)	<0.01	1.64	(1.48–1.81)	<0.01
Light drinkers (less than 4 drinks/day)		–			1.00			1.00		

Notes: Light Drinker: 4 Drinks/day or less; Heavy Drinker: 4 Drinks/day and more; K6: Kessler 6-item scale.

## Supplementary Materials

The following are available online at www.mdpi.com/1660-4601/14/10/1281/s1, Table S1. Multivariate logistic regression analysis of newly-started drinkers and continuing drinkers after having begun excepted 20–39 age category.
