# Supplementary material for: The Relationship between Starting to Drink and Psychological Distress, Sleep Disturbance after the Great East Japan Earthquake and Nuclear Disaster: The Fukushima Health Management Survey
Source: Int J Environ Res Public Health. 2017 Oct 24;14(10):1281. doi: 10.3390/ijerph14101281 (PMC5664781; doi:10.3390/ijerph14101281)
Supplement: Supplementary file 1 [file ijerph-14-01281-s001.pdf]

International Journal of
Environmental Research and Public Health

MDPI

Supplementary Materials: The Relationship between Starting to Drink and Psychological Distress, Sleep Disturbance after the Great East Japan Earthquake and Nuclear Disaster: The Fukushima Health Management Survey

Masatsugu Orui [1,*], Yuka Ueda [2], Yuriko Suzuki [1,3], Masaharu Maeda [4,6], Tetsuya Ohira [2,6], Hirooki Yabe [5,6] and Seiji Yasumura [1,6]

Table S1. Multivariate logistic regression analysis of newly-started drinkers and continuing drinkers after having begun excepted 20–39 age category.

		Sample 1 Newly–Started Drinkers vs. Continuing Non–Drinkers			Sample 2 Continuing Newly–Started Drinkers vs. Abstainers		
		Odds Ratio (95% CI)		*p* Value	Odds Ratio (95% CI)		*p* Value
**Gender**							
Male		2.14	(1.92–2.39)	<0.01	1.18	(0.89–1.55)	0.25
Female (Ref.)		1.00			1.00		
**Age (as of 11 March 2011)**							
40–64 years old		1.60	(1.42–1.80)	<0.01	1.39	(1.05–1.85)	0.02
65 years old and older (Ref.)		1.00			1.00		
**Education**							
Jurinor/ Senior high school		0.96	(0.93–0.99)	0.02	0.98	(0.91–1.06)	0.57
Vocational college, University, Graduated school (Ref.)		1.00			1.00		
**Subjective health condition**							
Not good		0.96	(0.93–0.99)	0.04	0.95	(0.87–1.04)	0.25
Good/ Unremarkable (Ref.)		1.00			1.00		
**Sleep disturbance**							
Unsatisfied with sleep condition	Yes	1.06	(1.03–1.10)	<0.01	1.03	(0.96–1.11)	0.44
	No	1.00			1.00		
**Psychological distress**							
K6 (13 points and more)	13≤	1.10	(1.06–1.14)	<0.01	1.06	(0.95–1.19)	0.27
	≤12	1.00			1.00		
**Disaster–related experience**							
Experience of tsunami	Yes	1.02	(0.99–1.06)	0.21	1.04	(0.96–1.13)	0.32
	No	1.00			1.00		

Experience of nuclear power plant accident	Yes	1.02	(0.99–1.05)	0.17	1.05	(0.95–1.16)	0.37
	No	1.00			1.00		
**Disaster effects on economic status**							
Loss of employment due to disaster	Yes	1.01	(0.98–1.04)	0.64	0.96	(0.89–1.03)	0.24
	No	1.00			1.00		
**Perception of radiation risks**							
Delayed effects	High	1.04	(1.01–1.07)	<0.01	1.07	(0.99–1.14)	0.06
	Low	1.00			1.00		
**Drinking behavior in 2012**							
Heavy drinkers (4 drinks/day and more)		–	–	–	1.30	(1.17–1.50)	<0.01
Light drinkers (less than 4 drinks/day)		–			1.00		

© 2017 by the authors. Submitted for possible open access publication under the terms and conditions of the Creative Commons Attribution (CC BY) license (http://creativecommons.org/licenses/by/4.0/).
